# Diagnostic Accuracy of Aldosterone and Renin Measurement by Chemiluminescence for Screening of Patients with Primary Aldosteronism

**DOI:** 10.3390/ijms25158453

**Published:** 2024-08-02

**Authors:** Martina Tetti, Jacopo Burrello, Jessica Goi, Mirko Parasiliti-Caprino, Giulia Gioiello, Fabio Settanni, Silvia Monticone, Paolo Mulatero, Giulio Mengozzi

**Affiliations:** 1 Division of Internal Medicine and Hypertension, Department of Medical Sciences, University of Torino, 10126 Torino, Italy; martina.tetti@unito.it (M.T.); jacopo.burrello@unito.it (J.B.); jessica.goi@unito.it (J.G.); silvia.monticone@unito.it (S.M.); 2 Division of Endocrinology, Diabetes and Metabolism, Department of Medical Sciences, University of Torino, 10126 Torino, Italy; mirko.parasiliticaprino@unito.it; 3 Clinical Biochemistry Laboratory, Department of Laboratory Medicine, University of Torino, 10126 Torino, Italy; giulia.gioiello@unito.it (G.G.); fabio.settanni@unito.it (F.S.); giulio.mengozzi@unito.it (G.M.)

**Keywords:** primary aldosteronism, aldosterone, direct renin, plasma renin activity, chemiluminescence, radio-immunoassay

## Abstract

Primary aldosteronism (PA) is the most common cause of endocrine arterial hypertension, and the suggested screening test for case detection is the aldosterone-to-renin ratio (ARR) or aldosterone-to-direct renin ratio (ADRR) based on radio-immunoassay (RIA) and chemiluminescence assay (CLIA), respectively. The objective of our study was to evaluate the reliability of CLIA for aldosterone and renin measurement and the diagnostic performance of ADRR. A prospective cohort of 1110 patients referred to a single laboratory medicine center underwent measurement of aldosterone and direct renin concentration (DRC) by CLIA and measurement of aldosterone and plasma renin activity (PRA) by RIA. Of 1110 patients, 640 obtained a final diagnosis of hypertension, and 90 of these patients were diagnosed with PA. Overall, between-method correlation was highly significant for aldosterone concentrations (*R* = 0.945, *p* < 0.001) and less strong but significant for DRC/PRA (*R* = 0.422, *p* < 0.001). Among hypertensive patients, in PA cases, the areas under the receiver operator characteristics (ROC) curves were 0.928 (95% confidence interval 0.904–0.954) for ADRR and 0.943 (95% confidence interval 0.920–0.966) for ARR and were comparable and not significantly different. The highest accuracy was obtained with an ADRR cut-off of 25 (ng/L)/(mIU/L), displaying a sensitivity of 91% and a specificity of 85%. The chemiluminescence assay for aldosterone and DRC is a reliable method for PA diagnosis compared to the classical RIA method.

## 1. Introduction

The most common endocrine cause of arterial hypertension is primary aldosteronism (PA) [1,2,3,4]. The aldosterone excess is associated with an increased cerebro- and cardiovascular risk compared with subjects affected by essential hypertension [5,6,7,8,9,10,11,12]. Indeed, there is evidence of higher risk of ischemic stroke, arrhythmias, coronary arteries disease, metabolic syndrome, diabetes, and chronic kidney disease compared to patients affected by essential hypertension (EH) [8,9,12,13]. The correct and prompt diagnosis of primary aldosteronism is pivotal in order to start the proper treatment and reverse the excess risk due to hyperaldosteronism [9,12,13,14,15].

According to the latest PA guidelines, up to 50% of hypertensive patients should be screened for PA [16,17]. Indeed, among patients with hypertension, several subgroups of patients display high probability of PA, such as patients with grade 2–3 hypertension or resistant hypertension, spontaneous or diuretic-induced hypokalemia, adrenal incidentaloma, atrial fibrillation, or early-onset hypertension [18,19]. The most reliable screening test is considered to be the ratio between serum/plasma aldosterone concentration (AC) and renin levels. Many confounding factors, such as drug therapies or potassium levels, could interfere with biochemical measurement [16,17,20,21,22]. Moreover, there are different laboratory methods for measuring aldosterone and renin, such as chemiluminescence-based immunoassay (CLIA) and radio-immuno assay (RIA). Due to the importance of testing conditions, the interfering drugs, and the lack of standardization among different measurement methods [16,17,23,24], the clinical practice guidelines indicate different ARR cut-offs that could be adopted [16,17,25,26].

Traditionally, RIA has been the routine assay used to measure AC and PRA [27,28], and it is very sensitive. Nevertheless, RIA is a manual and time-consuming method and produces radioactive waste that needs to be properly disposed. RIA measures aldosterone and plasma renin activity (PRA). PRA is calculated as the amount of angiotensin I produced from angiotensinogen present in the plasma sample as a function of time. In recent decades, CLIA has rapidly replaced other measurement methods in many centers due to several advantages [29,30], namely that CLIA can directly measure AC and direct renin concentration (DRC) on an automated platform, and is simpler in its handling of the plasma sample, and is a more time- and cost-effective method than RIA. The screening test performed with RIA examines the AC-to-PRA ratio (ARR, aldosterone-to-renin ratio) and with CLIA examines the AC-to-DRC ratio (ADRR, aldosterone-to-direct renin ratio). Recently, AC was also measured by liquid chromatography–tandem mass spectrometry (LC-MS/MS), which is considered to be the most accurate method; however, even with this technique, a high variability has been observed [31]. Moreover, this method is highly costly and is used in relatively few laboratories [32,33].

There are several studies [34,35,36,37] which have demonstrated that ARR and ADRR are both reliable and reproducible if performed under standardized conditions. Over recent years, CLIA has become available in many laboratories and preferred to RIA. In the literature, many studies have analyzed the accuracy and reproducibility of these hormonal assays in relatively small cohorts of patients and have yielded discordant results [31,34,38,39,40,41,42]. The aim of this study was to compare the measurement of aldosterone concentration and PRA/DRC using the CLIA and RIA methods and to compare the diagnostic accuracy of ADRR and ARR as screening for primary aldosteronism in a large prospective cohort of patients referred to the Department of Laboratory Medicine of a tertiary-level referral center.

## 2. Results

### 2.1. Characteristics of the Population

This study enrolled a total prospective cohort of 1110 patients referred for measurement of renin and/or aldosterone. Their clinical and biochemical characteristics are summarized in Table 1. The mean age of the cohort was 52 years, and there was a balanced representation of male (48.3%) and female (51.7%) patients.

For 640 out of the 1110 patients, it was possible to retrieve the final diagnosis. Of these, 550 (85.9%) had a final diagnosis of EH, and 90 (14.1%) had a diagnosis of PA. Among the PA patients, 24 (26.7%) were diagnosed with aldosterone-producing adenoma (APA), 34 (37.7%) were diagnosed with bilateral PA (BiPA), and for 32 (35.6%) patients, the subtype diagnosis was undetermined.

### 2.2. Comparison between Aldosterone Concentrations Measured by Radioimmunometric and Chemiluminescent Assay

**Figure 1:**
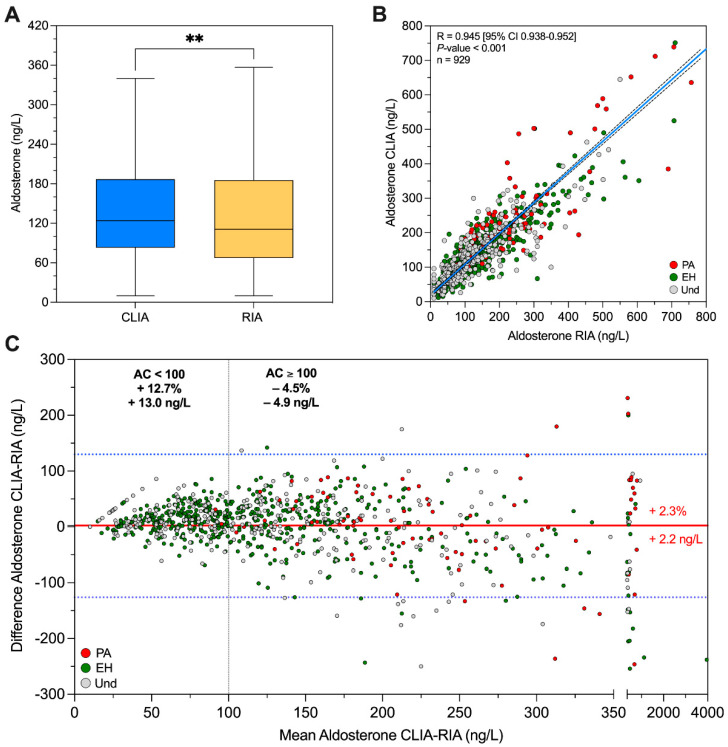
(**A**) Aldosterone concentration levels measured using the CLIA and RIA methods. Aldosterone concentration expressed in ng/L. ** *p* < 0.01 (**B**) Aldosterone concentration measured by CLIA and RIA correlation analysis assessed by Pearson’s R test. The *x*−axis shows AC by RIA (ng/L), while the *y*-axis shows AC by CLIA (ng/L). Green dot: patients with EH; red dot: patients with PA; grey dot: patients without a defined diagnosis; dashed lines: 95% confidence interval; continuous line: regression curve (Y = 0.89 × X + 19.03). (**C**) Bland−Altman plot for aldosterone concentration by CLIA vs. RIA. The *x*−axis shows the mean AC measurement by CLIA and RIA assays; the *y*−axis shows the difference between the AC measurements taken by CLIA and RIA assays. Green dot: patients with EH; red dot: patients with PA; grey dot: patients without a defined diagnosis. Continuous red line represents mean difference between AC measurement by CLIA and RIA; blue dashed lines represent 95% confidence interval. AC, aldosterone concentration; CLIA, chemiluminescence; EH, essential hypertension; PA, primary aldosteronism; RIA, radio−immunoassay; Und, undefined diagnosis.

In our cohort, the median aldosterone concentration was 124 ng/L [83; 187], as measured by CLIA; meanwhile, the median aldosterone concentration was 111 ng/L [68; 186], as determined by RIA (*p* < 0.01) (Figure 1A).

In the overall samples (n = 929), the correlation of AC between RIA and CLIA was highly significant (*R* = 0.945 [95% CI 0.938–0.952]; *p* < 0.001) (Figure 1B). If we considered only samples with AC ≥ 100 ng/L (n = 519), the correlation was *R* = 0.942 (*p* < 0.001) (Figure S1B), whereas for samples with AC < 100 ng/L (n = 410), the correlation was *R* = 0.656 (*p* < 0.001) (Figure S1A). Regression line equations are reported in the legend of Figure 1 and in Table S1.

According to Bland–Altman analysis, in the overall samples (n = 929), the mean difference in absolute AC between CLIA and RIA was 2.2 (±2.15) ng/L (Figure 1C). When considering only patients with AC ≥ 100 ng/L, the mean difference showed a negative trend (–4.9 ± 3.45 ng/L). Meanwhile, if we took into consideration only patients with AC < 100 ng/L, the mean difference between CLIA minus RIA was 13.0 (± 1.26) ng/L (Figure 1C). Overall, there is a mean 2.3% overestimation of aldosterone concentration by CLIA. For AC values ≥ 100 ng/L, CLIA shown an underestimation of 4.5% of AC. In particular, RIA gave higher values in samples with AC > 186 ng/L (IV quartile) (Table S2, Figure S2). For AC values < 100 ng/L, CLIA demonstrated an overestimation of 12.7% when compared with RIA (Table S2, Figure S2).

### 2.3. Comparison between Plasma Renin Activity and Direct Renin Concentration

**Figure 2:**
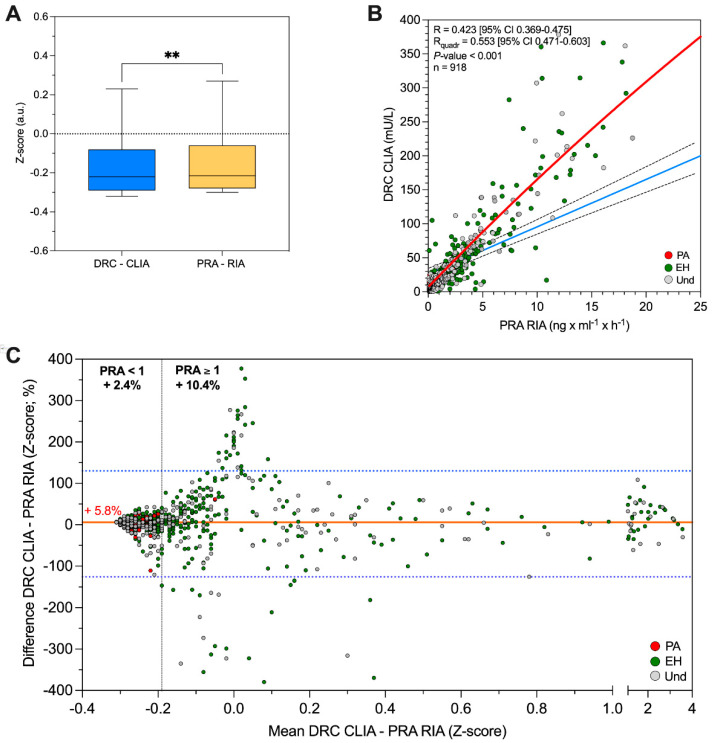
(**A**) DRC levels measured by CLIA and PRA levels measured by RIA. DRC and PRA levels are reported after normalization by Z-score (see methods). ** *p* < 0.01. (**B**) DRC by CLIA and PRA by RIA correlation analysis assessed by Pearson’s R test. The *x*-axis shows the PRA by RIA (reported as ng × mL^−1^ × h^−1^), while the *y*−axis shows the DRC by CLIA (reported as mIU/L). Green dot: patients with EH; red dot: patients with PA; grey dot: patients without a defined diagnosis; dashed lines: 95% confidence interval; continuous blue line: regression curve (Y = 6.97 × X + 25.88); continuous red line: quadratic fit line (Y = 6.9 + 16.6 × X − 0.07 × X^2^). (**C**) DRC by CLIA vs. PRA by RIA Bland–Altman plot. DRC and PRA are reported after normalization by Z−score. The *x*−axis shows the mean measurement of the DRC by CLIA and PRA by RIA assays; the *y*−axis shows the difference between the measurement of DRC by CLIA and PRA by RIA assays. Green dot: patients with EH; red dot: patients with PA; grey dot: patients without a defined diagnosis. Continuous red line represents the mean difference between measurement of DRC by CLIA and PRA by RIA; blue dashed lines represent 95% confidence interval. CLIA, chemiluminescence; DRC, direct renin concentration; EH, essential hypertension; PA, primary aldosteronism; PRA, plasma renin activity; RIA, radio−immunoassay; Und, undefined diagnosis.

In our cohort, the median DRC was 13.5 [4.5; 33.0] mIU/L, while the median PRA was 0.78 [0.27; 2.06] ng × mL^−1^ × h^−1^ (*p* < 0.01) (Figure 2A).

In the overall samples (n = 918), the correlation between DRC measured by CLIA and PRA measured by RIA was significant (*R* = 0.423 [95% CI 0.369–0.475]; *p* < 0.001) (Figure 2B). If we took into account only samples with PRA ≥ 1 ng × mL^−1^ × h^−1^ (n = 394), then the correlation was higher, with *R* = 0.668 (*p* < 0.001) (Figure S1D), whereas for the samples with PRA < 1 ng × mL^−1^ × h^−1^ (n = 524), there was no correlation (*R* = 0.026; *p* = 0.558) (Figure S1C). Thus, in the range of PRA < 1 ng × mL^−1^ × h^−1^, the regression line is flat. For this reason, we added a quadratic fit line to Figure 2B, an expression of a non-linear correlation for PRA and DRC. Regression line and quadratic regression equations are reported in the legend of Figure 2 and in Table S1. The same results were confirmed by the correlation analysis of PRA vs. DRC after normalization by Z-score (Table S1, Figure S3). Regression lines equations are reported in Table S1.

As shown in Bland–Altman graphic representation of the Z-score, there was a mean 5.8% overestimation of DRC compared to PRA (Figure 2C).

### 2.4. Diagnostic Accuracy

Among patients with hypertension, we selected patients with AC levels > 100 ng/L, measured by CLIA or RIA, and we calculated the ADRR and the ARR. The cut-off levels used for positive PA screening were ADRR ≥ 20 (ng/L)/(mIU/L) and ARR ≥ 300 ng/L/ng mL^−1^ h^−1^.

**Figure 3:**
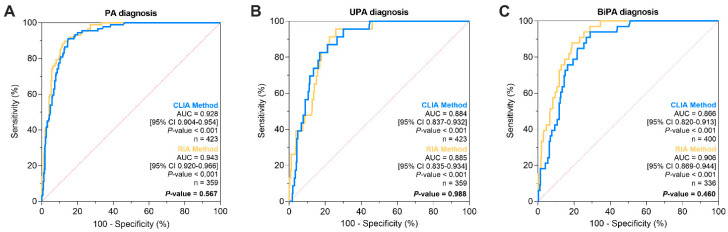
ROC curve analysis comparing ADRR as calculated using aldosterone and DRC by CLIA or ARR calculated using aldosterone and PRA by RIA for (**A**) patients with PA versus patients with EH and (**B**) UPA versus other patients with hypertension. (**C**) BiPA versus other patients with hypertension (UPA patients were not included in this analysis). The analysis was performed on patients with an AC ≥ 100 ng/L in CLIA or RIA, as appropriate. AUC by CLIA and RIA methods were compared in order to assess potential differences, and a *p*-value < 0.05 was considered significant. ADRR, aldosterone-to-direct renin ratio; ARR, aldosterone-to-renin ratio; AUC, area under the curve; BiPA, bilateral primary aldosteronism; CLIA, chemiluminescence; EH, essential hypertension; PA, primary aldosteronism; RIA, radio-immunoassay; UPA, unilateral primary aldosteronism; 95% CI, 95% confidence interval.

To assess the diagnostic accuracy of the two different assays, we used the receiver operator characteristics (ROC) curves (Figure 3). For patients with PA and EH, the areas under the curve (AUC) for the CLIA and RIA methods were not significantly different, with values of 0.928 (95% CI 0.904–0.954) and 0.943 (95% CI 0.920–0.966), respectively (Figure 3A). For PA diagnosis, ADRR showed the highest accuracy according to the Youden index with a threshold of 25 (ng/L)/(mIU/L), with a sensitivity of 91.1% and a specificity of 85.3%. For the ARR, the highest accuracy was reached with a cut-off of 436 ng/L/ng mL^−1^ h^−1^ with a sensitivity of 89.7% and a specificity of 87.1% (Table S3).

If we considered patients with a subtype diagnosis of UPA or BiPA, the AUC for the CLIA and RIA methods were not significantly different, showing a comparable diagnostic accuracy (Figure 3B,C). Different cut-off levels with relative sensitivity and specificity are given in Supplemental Table S3.

## 3. Discussion

In this study, we aimed to evaluate the correlation of aldosterone and renin measurements taken using the CLIA compared to the RIA method and the respective diagnostic accuracy in a large prospective cohort of 1110 patients referred to a tertiary laboratory medicine center. We observed a very good correlation between AC measured by CLIA and RIA in the overall samples, as reported in the literature [39,40,41,42,43]. In samples with aldosterone concentrations in the normal–high range, the correlation was high. Meanwhile, the correlation was weaker but still significant at the lower end of aldosterone concentration (<100 ng/L). When measured by CLIA, aldosterone concentration showed an overall overestimation of 2.3%. A major overestimation was demonstrated for low aldosterone levels (<100 ng/L), with a mean bias of 12.7%, as observed by others [41]; by contrast, for high values of aldosterone, we observed an underestimation of aldosterone levels by CLIA. The weaker correlation of aldosterone in the range < 100 ng/L could be an issue for the interpretation of saline infusion confirmatory tests because the recommended cut-off for AC is included in this range [32,44]. We previously observed a high concordance of 85% between CLIA and RIA in patients who underwent confirmatory testing. All patients with discordant results showed a mild phenotype of autonomous aldosterone production in the area of overlap between PA and low-renin essential hypertension [39]. Other studies confirmed the accuracy of the CLIA method during confirmatory tests in a Chinese population of patients with hypertension [45].

Some authors suggested measuring AC after confirmatory tests by LC-MS/MS [31]. However, the LC-MS/MS method is scarcely diffused, requires specific sample processing and specialized laboratory staff, and is more time-consuming and expensive [46,47]. Furthermore, the difference between CLIA and RIA in the low range of AC is nonetheless relatively small, and higher cut-offs for confirmatory testing have been proposed [32] to address this issue. In particular, a cut-off of 78 ng/L displayed a specificity of 87%, and none of the patients with unilateral PA were missed, together with a sensitivity of 86%. A cut-off of 62 ng/L provided a sensitivity of 95% with a specificity of 80% [32].

Since ARR is mathematically highly dependent on renin value, the presence of very low renin levels could result in high ARR even when plasma aldosterone is low. Thus, this explains our choice of a minimum AC that we fixed at > 100 ng/L. It should be noted that the choice of a minimum AC of 100 ng/L could potentially cause some patients with a very mild form of PA to be missed. For this reason, some authors consider the minimum AC for a positive screening test, as well as the cut-off for a positive saline suppression, to be 60 ng/L for the seated SST. In our experience, no patients with unilateral PA were observed in our unit with high ARR and AC between 60 and 100 ng/L. Therefore, at worst, we believe that we would miss a minor number of patients with bilateral forms of PA. However, we evaluated the number of patients with AC between 60 and 100 ng/L for both CLIA and RIA and a high ARR. We observed only 1 patient with AC measured by CLIA greater than 60 ng/L (AC of 98 ng/L) and 3 patients with AC measured by RIA (AC of 76, 89, and 93 ng/L). The impact of these patients on the ROC curves would be negligible.

Measurements of DRC and PRA in our cohort displayed a poor overall correlation. At a deeper level, we could not demonstrate a significant correlation between CLIA and RIA for PRA < 1 ng × mL^−1^ × h^−1^, as expected [39,42]. One possible cause is the relatively lower limit of RIA sensitivity. The discrepancy between DRC and PRA at low values could also partly be explained by a cross-reaction of circulating pro-renin in CLIA, which is 10- to 100-fold higher than active enzyme [35,48]. It has been demonstrated that a certain amount of exogenous pro-renin experimentally added to samples in a study caused a proportional increase in DRC measured by CLIA [35]. Therefore, in the lower range of renin concentration, the interference of pro-renin should be higher. Different inter-individual angiotensinogen levels [49] are another possible confounding factor that can affect PRA measurement and potentially reduce correlation between CLIA and RIA. Moreover, cryoactivation is another potential cause of overestimation of PRA and DRC in the assay, determining the conversion of pro-renin to renin not only at storage temperatures between −5 °C and +4 °C, but also in samples stored at −20 °C, as recently reported [50]. In our study, the temperatures that could cause potential cryoactivation were carefully avoided. On the other hand, samples with PRA ≥ 1 ng × mL^−1^ × h^−1^ showed a better correlation with DRC measurement. Despite these limitations, the ADRR displayed a similar accuracy of ARR for the detection of PA, and thus, the CLIA method can be reliably used for PA screening in patients with hypertension.

The Endocrine Society international guidelines recommend both ADRR calculated by CLIA measurements and ARR calculated by RIA measurements in order to detect PA patients [16]. Previous studies have compared the diagnostic performance of ADRR and ARR and demonstrated similar diagnostic accuracy [34,39,42] or the superiority of ADRR (to DRC measured by CLIA) [40]. In the present study, we demonstrated a good accuracy of ADRR measured by CLIA in the detection of both patients with PA and of patients with UPA. In our cohort, we observed higher accuracy with an ADRR cut-off of 25 (ng/L)/(mIU/L) (sensitivity 91%, specificity 85%). Given that PA case detection requires a high sensitivity, we suggest an ADRR between 20 and 25 (ng/L)/(mIU/L) [39,40]. Li et al. suggested an ADRR cut-off of 29.3 (ng/L)/(mIU/L), but this displayed a low sensitivity (80%) [42]. Instead, Manolopoulou et al. proposed a cut-off of 11.2 (ng/L)/(mIU/L) with a high sensitivity [38] but using a different CLIA method. In our cohort and with our CLIA method, the specificity of this cut-off would be too low. Given that the results demonstrated in our study could be applied for general PA screening, in the real clinical practice world, there are some cases, typically patients at high clinical risk without the possibility of a full and proper withdrawal of interfering medications, in which ARR may not be fully informative and the individual interpretation of aldosterone and renin are also required, preferably by an expert physician. The correlation equation provided in the present manuscript could be of help in the comparison of RIA and CLIA methods.

### Limitations

The main limitation of our study is the relatively low number of patients who were diagnosed with PA (14%), fewer of which with unilateral PA (4%). Nonetheless, the low number of PA patients is due to the recruitment of a large cohort (n = 1110) of unselected patients in the real-life daily practice of a single medicine laboratory. Thus, the low selection bias in our population supports the generalizability and reproducibility of our results.

A proportion of patients included in the present study did not obtain a diagnosis (PA or EH). The data from the aldosterone and PRA/DRC measurement of the whole cohort have been included for the correlation analysis. Only the data from patients who obtained a final diagnosis (PA or EH) were used for the diagnostic accuracy analysis.

In the PA diagnostic work-up, we used an ARR cut-off of ≥300 ng/L/ng mL^−1^ h^−1^ with a minimum aldosterone level of ≥100 ng/L, as is recommended by Endocrine Society guidelines [16] and commonly accepted in many international centers. However, it should be noted that these cut-offs could potentially miss some PA patients with very mild autonomous aldosterone secretion.

The aldosterone level cut-off for confirmation testing is not standardized and varies through different centers. In our center, we consider an aldosterone cut-off of 60 ng/L for seated SST. This aspect can have an impact on final diagnosis.

Among PA patients, 32 subjects did not obtain a subtype diagnosis and were classified as undetermined.

Another limitation is that we did not compare AC measured by CLIA and RIA with LC-MS/MS. LC-MS/MS is considered the most reliable method for aldosterone measurement, but it has shown considerable variability between laboratories [31], and it is still not FDA-approved. Furthermore, this technique is expensive and not widely available, so most laboratories are currently using aldosterone measurement with CLIA. Moreover, LC-MS/MS is a valid measurement method for aldosterone, but renin measurement is not available using this method.

Finally, due to the relatively low number of patients with reduced renal function in our cohort, it was not possible to evaluate the diagnostic accuracy of ADRR and ARR in these subjects.

## 4. Materials and Methods

### 4.1. Patient Selection

In this study, a total cohort of 1110 patients was analyzed. The patients were prospectively recruited between November 2020 and May 2021 at the Department of Laboratory Medicine (University of Torino) of Città della Salute e della Scienza University Hospital. Each patient included in the study underwent at least one measurement of aldosterone and/or renin. All biochemical measurements were performed at the Department of Laboratory Medicine (University of Torino). Aldosterone was assessed for 929 patients both by RIA and CLIA, PRA was assessed for 918 patients, and DRC was assessed for 920 patients.

Out of 1110 patients, 640 had a final diagnosis of EH or PA. All patients who obtained a final diagnosis (PA or EH) underwent the screening and confirmatory test without interfering medications, as recommended by international clinical practice guidelines for PA. The data of the other patients were used only for evaluating correlations between variables but not for diagnostic accuracy analysis. These patients were screened after the withdrawal of anti-hypertensive interfering medications for at least 3 weeks (for beta-blockers, angiotensin-converting enzyme inhibitors, and angiotensin receptor blockers) or 4 weeks (diuretics). Patients who were at risk for high blood pressure levels were treated with calcium channel blockers or alpha-blockers. The screening test was considered positive with an ARR ≥ 300 ng/L/ng mL^−1^ h^−1^ or ADRR ≥ 20 (ng/L)/(mIU/L) and aldosterone ≥ 100 ng/L [16,17]. Cases with suspected diagnosis of PA underwent confirmation/exclusion testing and subtype diagnosis according to the recommendations of the Endocrine Society Guidelines [16,17,51], as previously reported [39]. Briefly, patients with positive PA screening underwent a confirmation/exclusion test, intravenous seated saline load, or captopril challenge test (when patients were potentially at risk of volume expansion) according to guidelines recommendations [16,17]. Patients with confirmed PA underwent subtype diagnosis by adrenal computed tomography and adrenal venous sampling (AVS), according to international guidelines [16,17,51].

Informed consent was obtained from all subjects involved in the study.

### 4.2. Biochemical Measurements

Blood samples were collected in the morning, after the patients had been standing for at least 2 h and then had been seated before venipuncture for at least 15 min, centrifuged (3000 rpm, 15 min, room temperature), and handled at room temperature and then the plasma was immediately frozen at −80 °C, according to laboratory protocols. Samples for aldosterone were collected into serum separator tubes, centrifuged (3000 rpm, 15 min, room temperature) and handled at room temperature, and then the serum was immediately frozen at −80 °C. Immediately before the analysis, samples were rapidly thawed to room temperature and then appropriately processed according to the manufacturers’ instructions. Samples for PRA and DRC were thawed and analyzed simultaneously.

Angiotensin I RIA kit (Beckman Coulter, Inc., Brea, CA, USA) was used for measuring PRA according to the manufacturer’s instructions. Two aliquots of each sample were incubated, one at 4 °C and the other at 37 °C, and were then assayed for angiotensin I. PRA was calculated by subtracting the angiotensin I value measured at 4 °C from that measured at 37 °C in accordance with the incubation time. Within-run and between-run precision tests provided coefficient of variation ≤11.3% and ≤20.9%, respectively. The analytical sensitivity was 0.1 ng × mL^−1^ × h^−1^. Samples with values below analytical sensitivity were re-assayed after an incubation of 18 h.

The chemiluminescent immunometric method LIAISON (DiaSorin, Saluggia, Italy) applied to a fully automated analyzer was used for measuring DRC. Inter- and intra-assay variations were assessed using the two kit controls and two patient sample pools prepared in-house. Intra-assay variation was less than 7.2% over the range 25–107 mIU/L, and inter-assay variation was less than 10.4% over the range 4.9–110 mIU/L. The functional sensitivity was below 2.0 mIU/L, and the limit of detection was 0.33 mIU/L.

Radioimmunoassay ACTIVE Aldosterone RIA kit (Beckman Coulter, Brea, CA, USA) was used for measuring aldosterone concentration by RIA. Within-run and between-run precision tests provided coefficient of variation ≤4.5% and ≤9.8%, respectively.

A fully automated LIAISON aldosterone chemiluminescent immunoassay (DiaSorin, Saluggia, Italy) was used for measuring aldosterone concentration by CLIA. Pool samples and controls over the range 68–749 ng/L yielded intra- and inter-assay coefficient of variations of 1.8–4.2% and 5.6–10.5%, respectively. This assay had a wide measurement range varying from 9.7 ng/L up to 1000 ng/L. The functional sensitivity was 19.7 ng/L.

Patients were recruited prospectively, and RIA and CLIA samples were handled similarly, so no differences between methods could be attributed to different handling of the sample, such as freezing and thawing.

### 4.3. Statistical Analysis

Statistics were performed using IBM SPSS Statistics 25 (IBM Corp., Armonk, NY, USA) and GraphPad PRISM 9 (La Jolla, San Diego, CA, USA). Variable distribution was assessed by Kolmogorov–Smirnov test: normally-distributed variables (age) were expressed as the mean (m) ± standard deviation (SD), non-normally distributed variables (PRA, DRC, aldosterone, and ARR) were expressed as the median and interquartile range and analyzed by Mann,–Whitney test. PRA and DRC are reported also after normalization by Z-score according to the following equation: Z = (X − m)/(SD). Categorical variables (sex) were expressed as the absolute number and percentage. Correlations between RIA and CLIA measurements were assessed by Pearson’s *R* test and analysis of linear regression curves or quadratic regression curves when appropriate. Bland–Altman plots were used to evaluate the within-patient variability (RIA vs. CLIA measurements), and to detect systematic/proportional errors or magnitude dependent bias. The diagnostic accuracy of ARR (calculated using aldosterone and PRA by RIA vs. aldosterone and DRC by CLIA) was evaluated using receiver operator characteristics (ROC) curves. The Youden index was used to determine the cut-off with the highest accuracy. *p*-value less than 0.05 were considered significant.

## 5. Conclusions

In summary, our study demonstrated and confirmed a comparable diagnostic performance for the case detection of PA through ADRR based on DRC and aldosterone both measured by CLIA in a large cohort of patients when compared to validated ARR based on the classical RIA method. We showed a good correlation between AC measured by CLIA and RIA and between DRC and PRA. Samples with low aldosterone levels (<100 ng/L) displayed an acceptable correlation, even if it was lower than in patients with higher AC. We confirmed the lack of correlation between DRC and PRA for low renin values. Our results are of clinical importance for PA case detection in patients with hypertension.

## Figures and Tables

**Table 1:** Cohort characteristics. Data are shown as the mean ± standard deviation or median [25th–75th percentile] or absolute number (percentage), as appropriate. PA, primary aldosteronism; EH, essential hypertension; UPA, unilateral primary aldosteronism; BiPA, bilateral primary aldosteronism; RIA, radio-immunoassay; PRA, plasma renin activity; AC, aldosterone concentration; ARR, aldosterone-to-renin ratio; CLIA, chemiluminescence; DRC, direct renin concentration; ADRR, aldosterone-to-direct renin ratio.

Variable	N	Study Cohort
Age (years)	1110	52 ± 17.9
Sex (ref. female; n, %)	1110	574 (51.7)
Diagnosis PA (n, %) EH (n, %)	640	90 (14.1) 550 (85.9)
PA subtype Undetermined (n, %) UPA (n, %) BiPA (n, %)	90	32 (35.6) 24 (26.7) 34 (37.7)
RIA Method PRA (ng × mL^−1^ × h^−1^) AC (ng/L) ARR (ng/L/ng mL^−1^ h^−1^)	918 929 669	0.78 [0.27; 2.06] 111 [68; 186] 141.5 [64.3; 382.7]
CLIA Method DRC (mIU/L) AC (ng/L) ADRR [(ng/L)/(mIU/L)]	920 929 671	13.5 [4.5; 33.0] 124 [83; 187] 9.2 [4.4; 24.9]

## Data Availability

The raw data supporting the conclusions of this article will be made available by the authors on request.

## Supplementary Materials

The following supporting information can be downloaded at: https://www.mdpi.com/article/10.3390/ijms25158453/s1.
