# Supplementary material for: Diagnostic Accuracy of Aldosterone and Renin Measurement by Chemiluminescence for Screening of Patients with Primary Aldosteronism
Source: Int J Mol Sci. 2024 Aug 2;25(15):8453. doi: 10.3390/ijms25158453 (PMC11313086; doi:10.3390/ijms25158453)

Supplementary materials

Diagnostic accuracy of aldosterone and renin measurement by chemiluminescence for screening of patients with primary aldosteronism

Martina Tetti [1], Jacopo Burrello [1], Jessica Goi [1], Mirko Parasiliti-Caprino [2], Giulia Gioiello [3], Fabio Settanni[3], Silvia Monticone [1], Paolo Mulatero [1,*] and Giulio Mengozzi [3]

1 - Division of Internal Medicine and Hypertension, Department of Medical Sciences, University of Torino, 10126, Torino, Italy.
2 - Division of Endocrinology, Diabetes and Metabolism, Department of Medical Sciences, University of Torino, 10126 Torino, Italy.
3 - Clinical Biochemistry Laboratory, Department of Laboratory Medicine, University of Torino, 10126 Torino, Italy.

Contents

Table S1: Correlation analysis for DRC by CLIA *vs* PRA by RIA and aldosterone by CLIA *vs* RIA measurements

Table S2: Bland-Altman analysis: CLIA versus RIA measurement

Table S3: Diagnostic performance of ADRR *vs* ARR

Figure S1: Correlation sub-analyses for aldosterone and PRA/DRC

Figure S2: Bland-Altman sub-analyses for aldosterone and PRA/DRC

Figure S3: Correlation sub-analyses for PRA/DRC after normalization by Z-score

Table S1. Correlation analysis for DRC by CLIA *vs* PRA by RIA and aldosterone by CLIA *vs* RIA measurements. PRA and DRC are reported as international unit (SI) and after normalization by Z-score. The table reports sample size (N), Pearson's coefficient (R), and the equation of regression line. *Quadratic fit-line R value and equation line. Sub-analyses were performed for PRA lower and equal or higher than 1 ng x ml^{-1} x h^{-1}, and for aldosterone (RIA) lower and equal or higher than 100 ng/L. *P*-value < 0.05 were considered significant. AC, aldosterone concentration; CLIA, chemiluminescence; DRC, direct renin concentration; RIA, radio-immuno assay.

Variable		N	R	*P*-value	Regression line
**All data**					
DRC (CLIA) vs. PRA (RIA)	Z-score	918	+ 0.422	**<0.001**	Y = 0.42*X – 0.001
	SI		+ 0.423	**<0.001**	Y = 6.97*X + 25.88
			+ 0.553*	**<0.001**	$Y = 6.9 + 16.6*X – 0.07*X^2$
AC (CLIA) vs. AC (RIA) – ng/L		929	+ 0.945	**<0.001**	Y = 0.89*X + 19.03
**PRA < 1 ng x ml^{-1} x h^{-1}**					
DRC (CLIA) vs. PRA (RIA)	Z-score	524	– 0.018	0.677	Y = – 0.58*X – 0.37
	SI		– 0.026	0.558	Y = – 13.89*X + 20.33
**PRA ≥ 1 ng x ml^{-1} x h^{-1}**					
DRC (CLIA) vs. PRA (RIA)	Z-score	394	+ 0.668	**<0.001**	Y = 0.39*X + 0.15
	SI		+ 0.668	**<0.001**	Y = 6.35*X + 47.29
**Aldosterone (RIA) < 100 ng/L**					
AC (CLIA) vs. AC (RIA) – ng/L		410	+ 0.656	**<0.001**	Y = 0.96*X + 22.01
**Aldosterone (RIA) ≥ 100 ng/L**					
AC (CLIA) vs. AC (RIA) – ng/L		519	+ 0.942	**<0.001**	Y = 0.91*X + 9.68

Table S2. Bland-Altman analysis for DRC by CLIA *vs* PRA by RIA and aldosterone by CLIA *vs* RIA measurements. PRA and DRC are reported after normalization by Z-score. The table reports sample size (N), mean value for the *x*-axis (Z-score for PRA/DRC, and mean of aldosterone measured by CLIA and RIA), mean difference (reported as absolute value, n, or as percentage, %). Sub-analyses were performed for PRA lower and equal or higher than 1 ng x ml^{-1} x h^{-1}, for aldosterone (RIA) lower and equal or higher than 100 ng/L, and for quartiles of PRA/DRC and aldosterone.AC, aldosterone concentration; CLIA, chemiluminescence; DRC, direct renin concentration; N.A., not available; RIA, radio-immuno assay.

Variable	N	Mean (x-axis)	*Mean Difference (n)* (y-axis)	*Mean Difference (%)* (y-axis)
**All data**				
DRC (CLIA) vs. PRA (RIA) – Z score	918	– 0.22	N.A.	+ 5.8± 4.10
AC (CLIA) vs. AC (RIA) – ng/L	929	+ 157	+ 2.2± 2.15	+ 2.3± 1.32
**PRA < 1 ng/mL/min (mean Z-score < -0.19)**				
DRC (CLIA) vs. PRA (RIA) – Z score	524	– 0.26	N.A.	+ 2.4± 0.72
**PRA ≥ 1 ng x ml^{-1} x h^{-1} (mean Z-score ≥ -0.19)**				
DRC (CLIA) vs. PRA (RIA) – Z score	394	+ 0.35	N.A.	+ 10.4± 9.54
**PRA/DRC Quartile (mean Z-score)**				
I Quartile (<–0.28)	202	– 0.30	N.A.	+ 4.7± 0.31
II Quartile (–0.28 - –0.21)	232	– 0.26	N.A.	+ 3.0 ± 0.99
III Quartile (–0.22 - –0.06)	250	– 0.17	N.A.	+ 0.2 ± 3.85
IV Quartile (≥-0.07)	234	+ 0.69	N.A.	+ 15.5 ± 15.54
**Aldosterone < 100 ng/L (mean CLIA/RIA)**				
AC (CLIA) vs. AC (RIA)	371	+ 65.2	+ 13.0± 1.26	+ 12.7± 2.00
**Aldosterone ≥ 100 ng/L (mean CLIA/RIA)**				
AC (CLIA) vs. AC (RIA)	558	+ 218.8	– 4.9± 3.45	– 4.5± 1.70
**Aldosterone Quartile (ng/L; mean CLIA/RIA)**				
I Quartile (< 76.3)	232	+ 51.7	+ 11.1 ± 1.47	+ 12.4 ± 2.78
II Quartile (76.3 – 119.0)	232	+ 97.1	+ 14.8 ± 1.96	+ 10.5 ± 2.04
III Quartile (120.0 – 185.0)	231	+ 149.0	+ 10.4 ± 3.05	+ 1.7 ± 2.41
IV Quartile (≥186.0)	234	+ 330.4	– 26.9 ± 7.30	– 15.1 ± 2.89

Table S3. Diagnostic performance of ADRR *vs* ARR. The table reports ROC curve analysis comparing ADRR calculated using aldosterone and DRC by CLIA and ARR calculated using aldosterone and PRA by RIA. Sample size (N; PA, UPA, and BiPA patients are reported for each analysis between square brackets), area under the curve (AUC), 95% confidence interval (95% CI) and asymptotical significance are shown together with cut-off corresponding to highest accuracy (according to Youden index*), highest sensitivity (#) and specificity (§). *P*-value < 0.05 were considered significant. ADRR, aldosterone-to-direct renin ratio; AC, aldosterone concentration; ARR, aldosterone-to-renin ratio; BiPA, bilateral primary aldosteronism; DRC, direct renin concentration; PA, primary aldosteronism; PRA, plasma renin activity; RIA, radio-immuno assay; UPA, unilateral primary aldosteronism.

	Variable	N	AUC	95% CI	*P*-value	*Cut-off*	Sens (%)	Spec (%)
**PA Patients**	ADRR (DRC – CLIA) (AC ≥ 100 ng/L)	423 [87]	0.928	0.904-0.954	**<0.001**	9#	100.0	53.6
						20	93.3	81.1
						25*	91.1	85.3
						27	86.5	87.1
						436§	3.4	100.0
	ARR (PRA – RIA) (AC ≥ 100 ng/L)	359 [89]	0.943	0.920-0.966	**<0.001**	200#	100.0	66.5
						300	93.1	78.7
						436*	89.7	87.1
						2600§	13.8	100.0
**UPA Patients**	ADRR (DRC – CLIA) (AC ≥ 100 ng/L)	423 [23]	0.884	0.837-0.932	**<0.001**	11#	100	55.3
						20	95.7	69.3
						27	87.0	75.0
						37*	86.9	78.8
						483§	4.3	100.0
	ARR (PRA – RIA) (AC ≥ 100 ng/L)	359 [23]	0.885	0.835-0.934	**<0.001**	200#	100.0	53.9
						300	95.7	65.2
						460*	95.7	74.1
						8000§	8.7	100.0
**BiPA Patients**	ADRR (DRC – CLIA) (AC ≥ 100 ng/L)	400 [33]	0.866	0.820-0.913	**<0.001**	8.5#	100.0	48.9
						17.5*	93.4	71.0
						20	87.9	74.3
						27	78.8	80.1
						436§	6.1	99.7
	ARR (PRA – RIA) (AC ≥ 100 ng/L)	336 [33]	0.906	0.869-0.944	**<0.001**	230#	100.0	65.4
						300	93.9	71.6
						472*	87.9	81.2
						2645§	15.2	99.7

Figure S1. Correlation sub-analyses for aldosterone and PRA/DRC assessed by Pearson's R test. Green dot: patients with EH; red dot: patients with PA; grey dot: patients without a defined diagnosis; dashed lines: 95% confidence interval; continuous line: regression curve. *P*-value < 0.05 was considered significant. (**A**) Aldosterone concentration by CLIA *vs* RIA in patients with an AC < 100 ng/L. (**B**) Aldosterone concentration by CLIA *vs* RIA in patients with an AC ≥ 100 ng/L. (**C**) DRC *vs* PRA in patients with a PRA < 1 ng x ml^{-1} x h^{-1}. (**D**) DRC *vs* PRA in patients with a PRA ≥ 1 ng x ml^{-1} x h^{-1}. AC, aldosterone concentration; CLIA, chemiluminescence; DRC, direct renin concentration; EH, essential hypertension; PA, primary aldosteronism; PRA, plasma renin activity; RIA, radio-immuno assay; Und, undefined diagnosis.

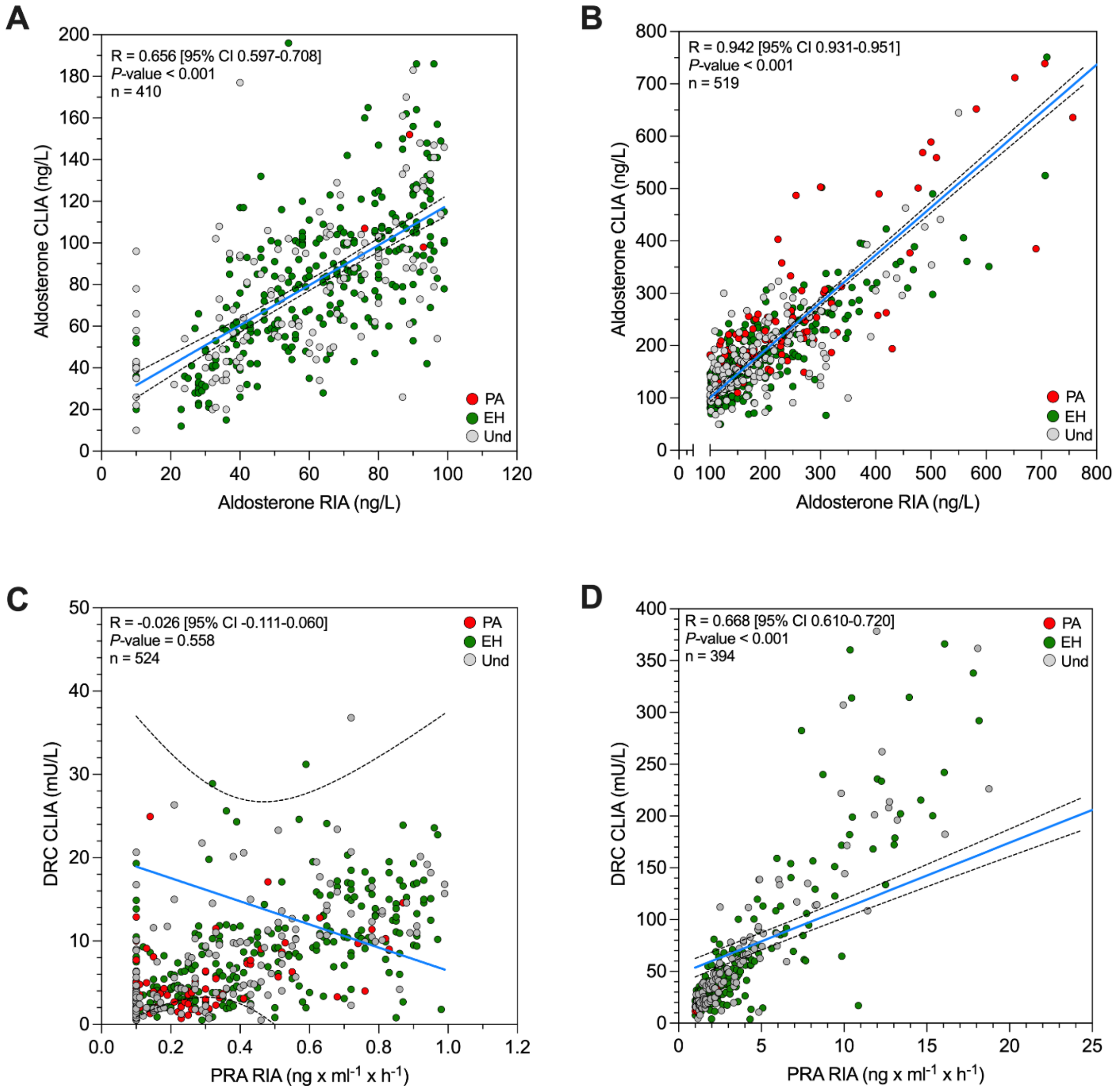

Figure S2. Bland-Altman sub-analyses for aldosterone and PRA/DRC. Green dot: patients with EH; red dot: patients with PA; grey dot: patients without a defined diagnosis. Continuous red line represents mean difference between measurement of DRC by CLIA and PRA by RIA; blue dashed lines represent 95% confidence interval. (**A**) On x-axis mean AC measurement by CLIA and RIA assays; on y-axis difference between AC measurement by CLIA and RIA assays. Aldosterone is reported in ng/L. (**B**) On *x*-axis mean measurement of DRC by CLIA and PRA by RIA assays; on *y*-axis difference between measurement of DRC by CLIA and PRA by RIA assays. DRC and PRA are reported after normalization by Z-score. AC, aldosterone concentration; CLIA, chemiluminescence; DRC, direct renin concentration; EH, essential hypertension; PA, primary aldosteronism; PRA, plasma renin activity; RIA, radio-immuno assay; Und, undefined diagnosis.

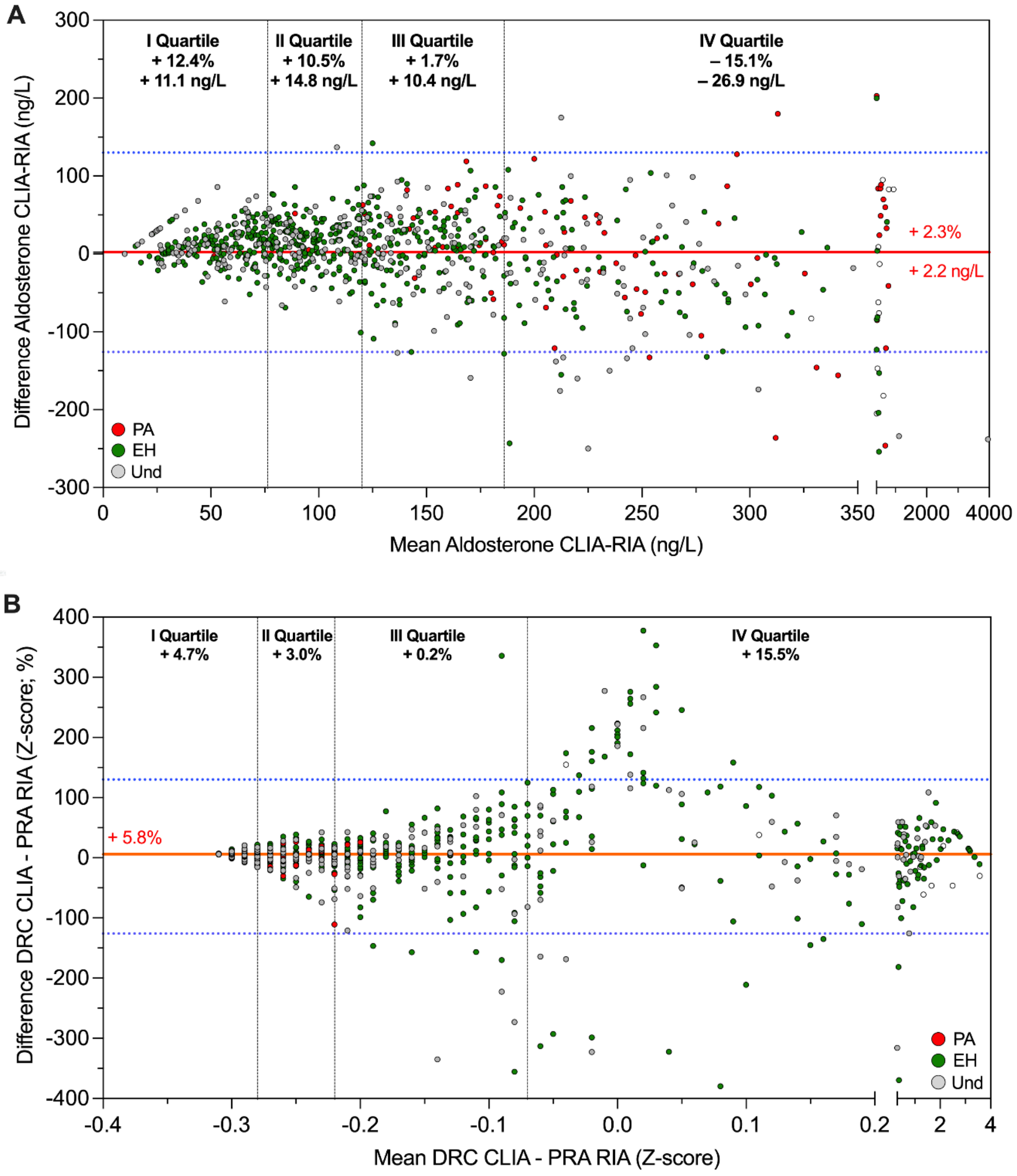

Figure S3. Correlation sub-analyses for PRA/DRC after normalization by Z-score assessed by Pearson's R test. Green dot: patients with EH; red dot: patients with PA; grey dot: patients without a defined diagnosis; dashed lines: 95% confidence interval; continuous line: regression curve. *P*-value < 0.05 was considered significant. (**A**) DRC *vs* PRA after normalization by Z-score. (**B**) DRC *vs* PRA in patients with a PRA < 1 ng x ml^{-1} x h^{-1} after normalization by Z-score. (**C**) DRC *vs* PRA in patients with a PRA ≥ 1 ng x ml^{-1} x h^{-1} after normalization by Z-score. CLIA, chemiluminescence; DRC, direct renin concentration; EH, essential hypertension; PA, primary aldosteronism; PRA, plasma renin activity; RIA, radio-immuno assay; Und, undefined diagnosis.

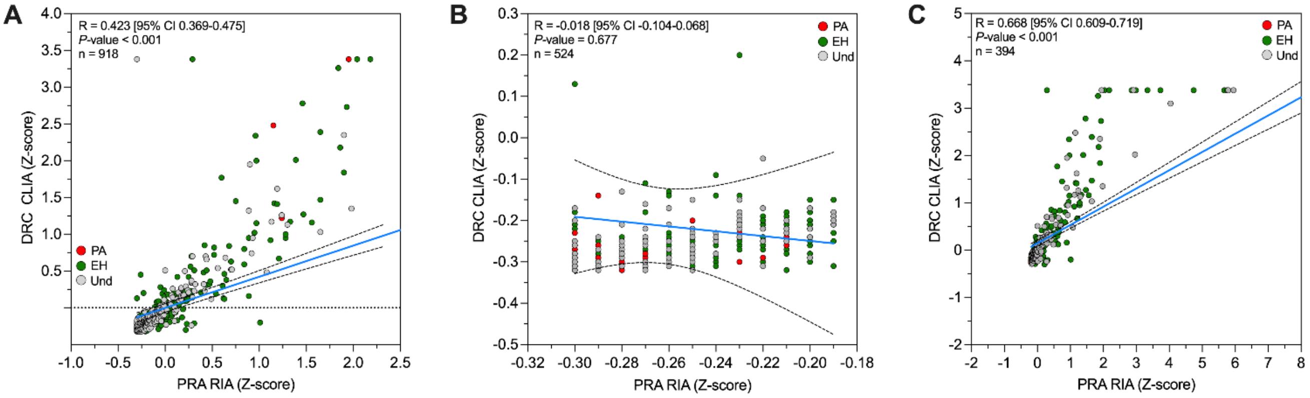

Supplement: Supplementary file 1 [file ijms-25-08453-s001.zip › ijms-3095006-supplementary.pdf]
